# Characterization of a Mouse-Adapted *Staphylococcus aureus* Strain

**DOI:** 10.1371/journal.pone.0071142

**Published:** 2013-09-02

**Authors:** Silva Holtfreter, Fiona J. Radcliff, Dorothee Grumann, Hannah Read, Sarah Johnson, Stefan Monecke, Stephen Ritchie, Fiona Clow, Christiane Goerke, Barbara M. Bröker, John D. Fraser, Siouxsie Wiles

**Affiliations:** 1 Department of Molecular Medicine and Pathology, University of Auckland, Auckland, New Zealand; 2 Department of Immunology, University of Greifswald, Greifswald, Germany; 3 Institute for Medical Microbiology and Hygiene, Technical University of Dresden, Dresden, Germany; 4 Alere Technologies, Jena, Germany; 5 Institute for Medical Microbiology and Hygiene, Universitätsklinikum Tübingen, Tübingen, Germany; University of Edinburgh, United States of America

## Abstract

More effective antibiotics and a protective vaccine are desperately needed to combat the ‘superbug’ *Staphylococcus aureus.* While in vivo pathogenicity studies routinely involve infection of mice with human *S. aureus* isolates, recent genetic studies have demonstrated that *S. aureus* lineages are largely host-specific. The use of such animal-adapted *S. aureus* strains may therefore be a promising approach for developing more clinically relevant animal infection models. We have isolated a mouse-adapted *S. aureus* strain (JSNZ) which caused a severe outbreak of preputial gland abscesses among male C57BL/6J mice. We aimed to extensively characterize this strain on a genomic level and determine its virulence potential in murine colonization and infection models. JSNZ belongs to the MLST type ST88, rare among human isolates, and lacks an *hlb*-converting phage encoding human-specific immune evasion factors. Naive mice were found to be more susceptible to nasal and gastrointestinal colonization with JSNZ than with the human-derived Newman strain. Furthermore, naïve mice required antibiotic pre-treatment to become colonized with Newman. In contrast, JSNZ was able to colonize mice in the absence of antibiotic treatment suggesting that this strain can compete with the natural flora for space and nutrients. In a renal abscess model, JSNZ caused more severe disease than Newman with greater weight loss and bacterial burden. In contrast to most other clinical isolates, JSNZ can also be readily genetically modified by phage transduction and electroporation. In conclusion, the mouse-adapted strain JSNZ may represent a valuable tool for studying aspects of mucosal colonization and for screening novel vaccines and therapies directed at preventing colonization.

## Introduction

The high incidence of illness and death within hospitals and the community caused by the bacterium *Staphylococcus aureus*, and the multidrug resistant nature of many isolates is reason for concern [1]–[3]. The spectrum of *S. aureus* disease ranges from skin and soft tissue infections and osteomyelitis to life threatening pneumonia and sepsis. Besides being a pathogen, *S. aureus* is also a frequent colonizer of the human mucosa and skin. About 20% of the healthy human population is persistently colonized in the nostrils, the throat or even the intestine [4]–[6], with the remainder being transiently colonized [7]. It is well established that mucosal colonization with *S. aureus* is major risk factor for the development of disease and transmission of bacteria to susceptible individuals [6], [8]. Nasal carriage can be transiently eradicated by topical administration of the antibiotic mupirocin but this is compromised by the development of resistance [9]. To develop alternative strategies for reducing nasal carriage, a detailed understanding of the molecular basis of interactions between the host and the bacterium that underlie the process is urgently required. Hence, there is a strong need for a robust and sustained mucosal colonization model that closely mimics the human clinical situation.

Mice are commonly used for staphylococcal colonization and infection models with the obvious advantages of having numerous gene knock-out strains at hand, a well-characterized immune system and being relatively easy and inexpensive to breed. However, whether mice are an appropriate model has often been questioned, based on the assumption that mice are not natural hosts of *S. aureus* [10].Furthermore, several virulence factors identified in human *S. aureus* isolates, such as Panton-Valentine leukocidin (PVL), Staphylococcal superantigen-like (SSL) proteins, and the phage-encoded immune evasion molecules staphylokinase (SAK), chemotaxis inhibitory protein (CHIPS) and staphylococcal complement inhibitor (SCIN), are human-specific and their study requires non-physiological concentrations in mouse models or do not work at all [11]–[14]. Thus, several aspects of human - *S. aureus* interactions cannot currently be modelled in the mouse.

The current dogma believed by the majority of *S. aureus* researchers is that mice are not natural hosts of *S. aureus* [10], [15]–[17], a misjudgement built on the lack of epidemiological data on *S. aureus* colonization and infection in laboratory rodents. In contrast, natural *S. aureus* colonization of laboratory mice, even under specific pathogen free (SPF) conditions, is relatively common, and the skin, the nasopharynx, and lower digestive tract have been identified as the characteristic ecological niches [18], [19]. Similar to humans, the hallmark of *S. aureus* infection in rodents is suppurative inflammation, with abscess formation in virtually any organ [18], [19].

We propose that the use of animal-adapted *S. aureus* strains could be a promising approach for developing clinically relevant animal infection models, because such strains should show enhanced fitness in their natural hosts when compared to human-adapted isolates. Recent genetic studies of *S. aureus* populations have demonstrated that *S. aureus* lineages are largely host-specific. For example, the majority of bovine and ovine mastitis are caused by a small number of host-specialized clones (CC97, CC151, and CC133), which are rarely isolated from humans [20], [21]. Similarly, the majority of *S. aureus* isolates from chickens belong to a poultry-adapted subtype of the worldwide human CC5-lineage [22], and ST398 is predominantly found in pigs [23], as well as in poultry [24]. However, to date a mouse-adapted *S. aureus* strain, which would be highly valuable for *S. aureus* research, has not been characterized.

In this study, we extensively characterized a mouse-adapted *S. aureus* strain, named JSNZ, with the goal of identifying features associated with adaption to the murine host. Furthermore, we have compared the ability of JSNZ to colonize and cause disease in mice relative to *S. aureus* strain Newman, a human isolate commonly used for mouse studies. We demonstrate that JSNZ belongs to the MLST type ST88, which is rare among human isolates, and lacks the human-specific hlb-converting phage. JSNZ also showed an enhanced ability for colonization and infection in the mouse and could be easily genetically modified.

## Materials and Methods

### *S. aureus* identification and DNA isolation

Murine *S. aureus* isolates were obtained from stool samples and preputial abscesses from mice bred in the animal facility at the University of Auckland. Human *S. aureus* isolates were obtained from a study of patients with bacteraemia admitted to hospital in Auckland. These patients provided written informed consent to characterize the isolates that caused their illness. *S. aureus* strain Newman was kindly provided by Eric Skaar.

*S. aureus* was identified by colony morphology on mannitol salt agar (MSA) plates, *S. aureus*–specific latex agglutination test (Staph Xtra Latex kit, ProLex™, Richmond Hill, ON, Canada) and gyrase PCR (see below). Total *S. aureus* DNA was isolated with the QIAGEN blood and tissue kit (QIAGEN).

### Genotyping of *S. aureus* strains

*Spa* genotyping and multilocus sequence typing (MLST) were performed as described elsewhere [9], [25].

### Virulence gene detection and phage typing

Multiplex PCRs were applied to detect a total of 25 *S. aureus* virulence genes, including gyrase (*gyr*), methicillin resistance (*mecA*), Panton-Valentine leukocidin (*pvl*), staphylococcal superantigens (*sea-selu, tst*), exfoliative toxins (*eta, etd*), *agr* group 1–4 and *S. aureus* bacteriophage types (Sa1int – Sa7int) as previously reported [26], [27]. An allelic variant of *ssl*11 found in JSNZ was detected using the following primers Ssl11_JSNZ_for (5′-GGGATCCAGCAGATTAGCAGTTACTTC-3′) and Ssl11_rev (5′-CGGAATTCTTATAAATTCACTTCAATTTT-3′), whereas ssl11 in Newman was detected using the forward primer Ssl11_for (5′-GGGATCCAGTACATTAGAGGTTAGATC-3′).

### Virulence gene detection by DNA microarray

The StaphID DNA microarray covers 333 target sequences, corresponding to 170 distinct *S. aureus* genes and their allelic variants, including species markers, resistance genes, exotoxins, adhesins, surface proteins, capsule synthesis genesand *agr* group typing markers. The principle of the assay, related procedures, and a list of targets has been described previously [28], and the system is commercially available (StaphyType by Alere Technologies GmbH, Jena, Germany).

### Screening stool samples for *S. aureus* JSNZ

Mouse stools (ca. 1 g) were collected from cages, homogenized in 1 ml sterile PBS, plated onto MSA plates and incubated over night at 37°C. Presumptive *S. aureus* colonies were sub-cultured onto MSA plates and subsequently tested with an *S. aureus*–specific latex agglutination test (Pro-Lab Diagnostics) and catalase test. *S. aureus* was differentiated from *S. sciuri* by an oxidase test (Microdase Disk, Remel, Dartford, Kent, UK). Finally, *S. aureus* colonies were analysed by a JSNZ colony multiplex PCR amplifying 16SrRNA, which served as quality control (756 bp; 16SrRNA_for 5′AACTCTGTTATTAGGGAAGAACA-3′, 16SrRNA_rev 3′-CCACCTTCCTCCGGTTTGTCACC-3′), *S. aureus*-specific gyrase (281 bp; Gyr_for 5′-AGTACATCGTCGTATACTATATGG-3′, Gyr_rev 5′-ATCACGTAACAGTTCAAGTGTG-3′), the phage integrase gene Sa3int, which is rare in animal-adapted strains (475 bp; Sa3int_for 5′- GAAAAACAAACGGTGCTAT-3′, Sa3int_rev 5′- TTATTGACTCTACAGGCTGA-3′) and the *ssl11_JSNZ* variant found in JSNZ and closely related CC88 strains (see above). *S. aureus* colonies were resuspended in 10 µl DNase and RNase free water and heat-inactivated for 10 minutes at 95°C. Multiplex PCRs were performed in 25 µl reactions containing 1× reaction buffer (50 mM KCl, 10 mM TrisHCl pH 9.0, 0.1% (v/v) Triton X-100), 3 mM MgCl_2_, 0.2 mM dNTPs, 0.1–0.3 µM of each primer, 2.5 U taq polymerase and 1 µl of the heat-inactivated *S. aureus* suspension. An initial denaturation of DNA at 95°C for 5 min was followed by 30 cycles of amplification (95°C for 45 s, 55°C for 45 s, and 72°C for 45 s), ending with a final extension phase at 72°C for 10 min.

### Animals

Female CD1 mice were obtained from the SPF breeding facility at the University of Auckland. The mice were 6 to 8 weeks of age upon arrival and were given food and water ad libitum. Animals were housed and cared for in accordance with The Animal Welfare Act (1999) and institutional guidelines provided by the University of Auckland Animal Ethics Committee, which reviewed and approved these experiments under applications R752 and R847. All mice were screened for *S. aureus* colonization prior to experimentation.

### Murine colonization model

To differentiate the inoculated *S. aureus* strains from the endogenous flora, *S. aureus* strains were marked with resistance to streptomycin (Sm) by selection of bacteria on TSA plates containing 0.5 mg/ml Sm (Sigma-Aldrich, Inc, St. Louis, MO, USA). To prepare the inocula, bacteria were grown to logarithmic growth phase in TSB medium (A_600_ of 0.5–0.7), pelleted and washed twice with sterile PBS pH 7.4. The total number of CFU in each inoculum was quantified by plating serial dilutions of the inoculum on TSA with Sm. In most experiments, 0.5 mg/ml Sm was added to the drinking water 5 days before inoculation to reduce the endogenous flora.

Female CD1 mice aged 8–10 weeks were intranasally inoculated with Sm-resistant (Sm^R^) *S. aureus* (10^7^ or 10^8^ CFU *S. aureus* in 10 µl of PBS) or PBS as a control as described by Kiser et al [29]. Bacterial loads were quantified in nasal tissue, lung, liver, kidneys, spleen, and cecum using an OMNI tissue homogenizer (OMNI International, Kennesaw, USA). GI colonization by *S. aureus* was verified by bacterial culture of ceca and feces. The cecum was opened longitudinally, rinsed with sterile PBS and homogenized. Cecum contents were suspended in 5 ml sterile PBS. Fresh feces were collected from each mouse, weighed, and suspended in PBS (0.1 g of feces per ml PBS). 25 ul of serial dilutions of homogenized organs, blood and feces or cecum suspensions were plated in triplicates on (1) selective plates containing 0.5 mg/ml Sm to determine the number of *S. aureus* CFU/g tissue and on (2) TSA plates without antibiotics to determine the total endogenous flora. The lower detection limits for the different murine samples were 133 CFU/g for stool samples, ∼10 CFU/g for noses, ∼88 CFU/g for spleen, 16.5 CFU/g for liver, 66 CFU/g for kidney, ∼15 CFU/g for cecum contents and ∼13 CFU/ml for abscesses. Culture negative samples were plotted at the detection limit.

### Murine renal abscess model

Female CD1 mice aged 7–8 weeks received ∼1.5×10^8^ CFU log-phase (A_600_ of 0.7) *S. aureus* JSNZ Sm^R^ and Newman Sm^R^ intraperitoneally to establish a systemic infection0. Animals were monitored daily for alterations in body weight and general health. Pre-determined animal welfare endpoints for these studies were >20% weight loss; >15% weight loss in a 24 h period; or two of the following signs of ill-health: ruffled fur, inactivity, hunched posture. Kidney, liver and spleen tissues were collected on day 4 for an assessment of bacterial load as described above.

### Murine subcutaneous abscess model

Log-phase (A_600_ of 0.5) *S. aureus* JSNZ Sm^R^ and Newman Sm^R^ were washed and diluted in PBS and then mixed 1∶1 in sterile cytodex bead (Sigma) solution (0.5 g/mL in PBS) as previously described [30]. Female CD1 mice aged 7–8 weeks were anaesthetized with isoflurane, the flank area shaved and ∼5×10^6^ CFU bacteria injected subcutaneously into the right and left flanks. Animals were monitored daily for alterations in body weight and general health. Abscess tissue was harvested from groups of animals on days 2 and 4 to determine *S. aureus* loads as described above.

### Statistics

Data analysis was performed as indicated in the figure legends using the GraphPadPrismX3 package.

## Results

### *S. aureus* caused an outbreak among C57BL/6J mice in an animal breeding facility

In September 2008, an outbreak of preputial gland infections was observed among a colony of C57BL/6J mice in the animal breeding facility of the University of Auckland. Mice were assessed by a veterinarian and abscesses were detected in 79 out of 370 animals. This outbreak particularly affected male C57BL/6J mice, which were strongly impaired in their mating performance, while females were generally symptom-free. Between October 2008 and February 2009, eight samples were collected from the preputial glands of infected mice for microbiological analysis. *S. aureus* was isolated from all samples in pure culture. In search of the origin of the *S. aureus* strain, animal-handling staff were screened for *S. aureus* nasal carriage. However, all colonized persons (2/13) carried strains that had different *spa* types (t012 and t081) to the murine isolates (*spa* type t729).

### Murine *S. aureus* strains are clonal and lack the *hlb*-integrating Sa3int phage

To determine whether the observed symptoms were caused by a single *S. aureus* clone, we performed *spa* and MLST typing. Genotyping demonstrated that all eight *S. aureus* isolates from infected C57BL/6J mice belonged to *spa* type t729 and MLST type ST88, suggesting a clonal outbreak (Table 1).

**Table 1:** Genotype, virulence genes and phages of murine and human CC88 isolates.

				Genotype			Virulence genes^1^								Phage families						
Name	Isolation date	Species	Type of infection	*spa* type	MLST ST	MLST CC	*nuc*	*mecA*	*ssl11_ JSNZ*	*ssl11*	*agr*	*non-egc SAgs*	*egc SAgs*	*eta, etd*	*Sa1int*	*Sa2int*	*Sa3int*	*Sa4int*	*Sa5int*	*Sa6int*	*Sa7int*
Mu#0	14.10.2008	male C57BL/6J mice	preputial gland abscess	t729	ST88	CC88	+	-	+	-	*3*	-	-	-	+	-	-	-	-	-	-
Mu#1	14.10.2008	male C57BL/6J mice	preputial gland abscess	t729	ST88	CC88	+	-	+	-	*3*	-	-	-	+	-	-	-	-	-	-
Mu#2 (JSNZ)	17.12.2008	male C57BL/6J mice	preputial gland abscess	t729	ST88	CC88	+	-	+	-	*3*	-	-	-	+	-	-	-	-	-	-
Mu#3	14.10.2008	male C57BL/6J mice	preputial gland abscess	t729	ST88	CC88	+	-	+	-	*3*	-	-	-	+	-	-	-	-	-	-
Mu#4	14.10.2008	male C57BL/6J mice	preputial gland abscess	t729	ST88	CC88	+	-	+	-	*3*	-	-	-	+	-	-	-	-	-	-
Mu#5	14.10.2008	male C57BL/6J mice	preputial gland abscess	t729	ST88	CC88	+	-	+	-	*3*	-	-	-	+	-	-	-	-	-	-
Mu#7	03.02.2009	male C57BL/6J mice	preputial gland abscess	t729	ST88	CC88	+	-	+	-	*3*	-	-	-	+	-	-	-	-	-	-
Mu#8	03.02.2009	male C57BL/6J mice	preputial gland abscess	t729	ST88	CC88	+	-	+	-	*3*	-	-	-	+	-	-	-	-	-	-
J10^2^	12.04.2011	C57BL/6J mice	colonization	t729	n.d.	CC88	+	-	+	-	*3*	-	-	-	+	-	-	-	-	-	-
J15^3^	12.04.2011	C57BL/6J mice	colonization	t729	n.d.	CC88	+	-	+	-	*3*	-	-	-	-	-	-	-	-	-	-
M3	02.05.2007	human	skin and soft tissue infection	t186	ST88	CC88	+	-	+	-	*3*	-	-	-	+	-	+	-	-	-	-
M25	22.08.2007	human	bacterial endocarditis	t186	ST88	CC88	+	-	+	-	*3*	-	-	*eta*	+	-	+	-	-	-	-
F25	01.04.2008	human	skin and soft tissue infection	t11192	ST88	CC88	+	-	+	-	*3*	-	-	-	-	-	+	-	-	-	+
A7	13.04.2007	human	intravenous device infection	t692	ST78	CC88	+	-	+	-	*3*	-	-	-	-	-	+	+	-	-	-
A50	15.08.2007	human	febrile neutropenia	t186	ST78	CC88	+	-	+	-	*3*	-	-	-	-	+	+	-	-	-	-
Newman	1952	human	infection	t008	ST8	CC8	+	-	-	+	*1*	*a*	-	-	-	-	+	-	+	+	+

1 *nuc*  =  nuclease, *mecA*  =  methicillin resistance, *ssl11*  =  staphylocococcal superantigen-like protein 11, *agr*  =  accessory gene regulator, SAg  =  superantigen, *egc*  =  enterotoxin gene cluster, *et*  =  exfoliative toxin.

2 isolated from a breeding pair held in individually ventilated cages in SPF facilities.

3 isolated from a breeding pair held in open-top cages in high containment area.

To elucidate what confers the host-specificity of the murine ST88 strains, we determined the virulence gene repertoire by multiplex PCR and compared it to five clinical human isolates: three ST88 isolates (M3, M25, and F25) and two closely related ST78 isolates (A7 and A50). All murine isolates showed an identical virulence gene pattern, corroborating their clonality (Table 1), whereas the gene pattern of the human isolates varied. Both murine and human ST88 strains were *agr3* positive and lacked superantigen genes, the *mecA* gene conferring methicillin resistance and *pvl* genes encoding the Panton-Valentine leukocidin (Table 1, 2). The murine ST88 and human ST78/88 strains also harboured an *ssl11* variant, called *ssl11_JSNZ* (NCBI accession number KC128615), which can be detected using specific primers.

**Table 2:** DNA microarray analysis of JSNZ and human CC88 isolates.

		JSNZ_ST88	M3_ST88	M25_ST88	F25_ST88	A50_ST78	A7_ST78
**Resistance Genotype**							
*MecA*	Methicillin resistance, defining MRSA	-	-	-	-	-	-
*blaZ, I, R*	Beta-Lactamase, Bla Repressor, Bla Regulatory Protein	-	+	+	-	+	+
*SCC*-type	Staphylococcal chromosomal cassette	-	-	-	-	-	-
**Virulence Genotype**							
*PVL*	Panton-Valentine leukocidin	-	-	-	-	-	-
*Hlb*	Haemolysine Beta	+	+	+	+	+	+
*hlb, untr.*	Haemolysine Beta (un-truncated)	+	-	-	-	+/−	+/−
*Sak*	Staphylokinase	-	+	+	+	+	+
*Chp*	Chemotaxis Inhibitory Protein (CHIPS)	-	-	-	-	-	+/−
*Scn*	Staphylococcal Complement Inhibitor (SCIN)	-	+	+	+	+	+
*EtA*	Exfoliative Toxin A	-	-	+	-	-	-
***agr*** ** Typing**		-	-	-	-	-	+/−
*AgrI*	Accessory Gene Regulator - Type 1						
*AgrII*	Accessory Gene Regulator - Type 2	-	-	-	-	-	-
*AgrIII*	Accessory Gene Regulator - Type 3	-	-	-	-	-	-
*AgrIV*	Accessory Gene Regulator - Type 4	+	+	+	+	+	+

By phage typing, all murine *S. aureus* isolates were found to carry a Sa1int phage, however only two out of five human strains carried this phage. Interestingly, all murine strains lacked an *hlb*-integrating Sa3int phage, which can encode the human-specific immune evasion factors SAK, CHIPS and SCIN [31]. This phage type was detected in all five human ST78/88 strains.

Finally, we carried out a panoramic comparison of the virulence gene repertoire of our murine ST88 and human ST78/88 isolates by genomic DNA microarray hybridization. Since all murine ST88 strains were clonal, we chose Mu#2, named hereafter JSNZ, as a representative and compared it to the five human ST78/88 strains. Overall, the microarray data confirmed the PCR data (Table 2, for complete data set refer to Table S1). In agreement with the absence of an Sa3int phage, JSNZ encoded a full-length *hlb* gene and lacked the phage-encoded immune evasion gene cluster. Conversely, the human Sa3int-phage positive isolates harboured a truncated *hlb* gene and carried *sak* and *scn*. JSNZ did not contain antibiotic resistance genes, whereas the human isolates encoded the beta-lactamase gene cluster. The novel *ssl11* variant described above could not be detected by the microarray targets, which flagged the gene as absent. Thus, with the exception of the Sa3int phage and beta-lactamase gene cluster, the murine ST88 and human ST78/88 isolates showed identical virulence gene patterns.

For completeness, we also compared JSNZ with *S. aureus* strain Newman, which was used as a reference strain in the mouse infection studies outlined below (Table S1). Newman belongs to *S. aureus* lineage CC8 and therefore displays the CC8 lineage-specific variants of surface proteins and regulatory genes. Moreover, it harbours four phages and carries the genes for a different capsule type. While Newman harbours the cap5 genes and produces a serotype 5 capsule, strain JSNZ carries the cap8 genes.

### JSNZ persisted within the C57BL/6J colony for 2.5 years, colonizing the murine nose and gastrointestinal tract

Once the outbreak of preputial gland abscesses among C57BL/6J mice declined, no further *S. aureus* infections were observed in the breeding facility for 2.5 years. To find out whether *S. aureus* JSNZ was still present, and whether it had spread to other colonies, we screened stool samples from cages of C57BL/6J (n = 24), CD1 (n = 7) and BALB/c (n = 6) breeders for *S. aureus* JSNZ by multiplex PCR. Notably, JSNZ was detected in 9 out of 24 C57BL/6J breeding pairs. Genotyping of two representative isolates obtained from breeding pairs held in individually ventilated cages within the specific pathogen free (SPF) breeding area (J10) and in an open-top cage in the high containment facility (J15), demonstrated the same *spa* type and MLST type (Table 1). Importantly, JSNZ was not found in CD1 and BALB/c mice bred in the same areas, demonstrating that good hygiene in animal facilities can prevent the spread of *S. aureus*.

To determine natural colonization patterns we culled five female and six male C57BL/6J mice derived from a JSNZ-colonized breeding pair and determined bacterial loads in the nose and caecal contents. Nine out of 11 mice were colonized with JSNZ and in most cases (8/9) the strain colonized both the murine nose and gastrointestinal (GI) tract. One mouse was colonized only in the nose at low bacterial density (data not shown). Bacterial loads on mannitol salt agar plates ranged from 10 to 3×10^4^ colony forming units (CFU)/g tissue in the nose (mean of 5.6×10^3^ CFU/g) and from 400 to 2×10^4^ CFU/ml in the caecum contents (mean of 1.2×10^3^ CFU/ml). However, a co-detection of other mannitol-fermenting bacteria, e.g. *S. sciuri* and *S. xylosus*, could not be excluded in this assay.

### JSNZ efficiently colonized mice and caused sustained systemic infection

Adaptation of JSNZ to the murine host should lead to enhanced fitness and/or virulence. Therefore, we determined the virulence potential of JSNZ in murine colonization and infection models. We used female CD1 mice for all experiments because these mice were not affected by the JSNZ outbreak. As a reference strain we used *S. aureus* strain Newman, which has a robust phenotype in murine infection models and is one of the human *S. aureus* strains most commonly used in animal infection studies.

**Figure 1:**
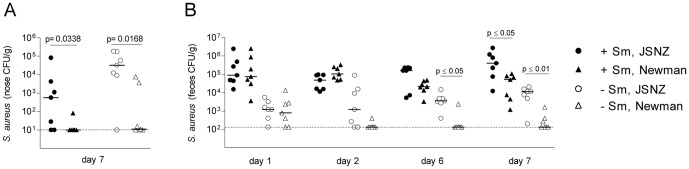
JSNZ efficiently colonizes the nose and gastrointestinal tract of CD1 mice. Female CD1 mice were intranasally inoculated with 10^8^ CFUs *S. aureus* JSNZ Sm^R^ and Newman Sm^R^. Mice were pre-treated with Sm to reduce the natural flora (filled symbols) or left untreated (empty symbols). Bacterial loads in the nose (A) and feces (B) were determined at indicated time points and the median is shown. Data were compared using a two-tailed Mann Whitney test (A, p = 0.0338 for Sm-treated JSNZ *versus* Newman and p = 0.0168 for Sm-untreated JSNZ *versus* Newman) or a Friedman test followed by Dunn's correction for multiple comparisons (B, p = 0.0455 for Sm-treated JSNZ *versus* Newman and p = 0.0003 for Sm-untreated JSNZ *versus* Newman). Culture negative samples were plotted at the detection limit (dashed line). One representative experiment out of two is shown.

Firstly, we investigated the dynamics of nasal colonization using an intranasal colonization model described by Kiser et al. We inoculated mice intranasally with 10^8^ CFU of spontaneously streptomycin resistant (Sm^R^) derivatives of *S. aureus* JSNZ or Newman. Mice were pre-treated with streptomycin (Sm) to reduce the natural flora, or left untreated. JSNZ efficiently colonized the nasal mucosa of both Sm treated (5/7) and untreated mice (6/7) (Fig. 1A). Since JSNZ naturally colonizes both nose and GI tract, we also collected stool samples from each mouse during the course of experimental colonization. Notably, JSNZ translocated from the nose to the GI tract within a day and persistently colonized the gut with high bacterial loads (Fig. 1B). While nasal colonization rates were comparable in Sm-treated and untreated mice, gut colonization was more efficient after antibiotic pretreatment (median 4×10^5^ CFU/g vs. 1.1×10^4^ CFU/g). In contrast, nasal colonization with *S. aureus* Newman was less successful. Only 1 out of 7 Sm-treated mice exposed to Newman remained colonized after 7 days. While JSNZ colonized the gut mucosa of both Sm-treated and untreated mice, Newman could establish itself only in the gut of Sm-treated mice.

In a follow-up experiment, JSNZ persistently colonized mice for up to 28 days with bacteria recovered from the noses of 5/7 mice and the GI tracts of 7/7 mice. In contrast, Newman was only recovered from the noses of 2/7 mice and the GI tracts of 3/7 mice (data not shown).

**Figure 2:**
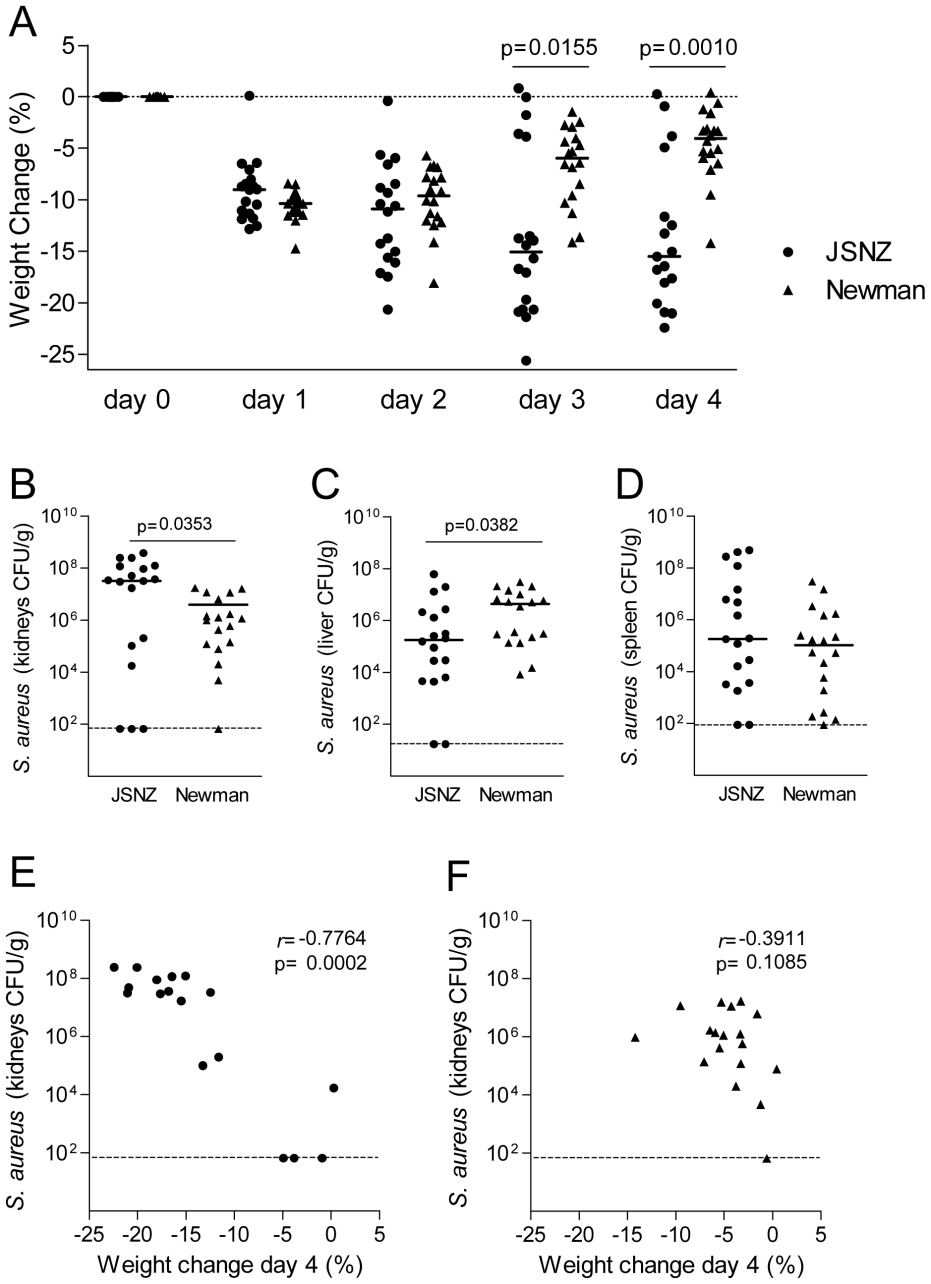
Systemic inoculation of mice with JSNZ causes sustained weight loss and enhanced renal abscess formation. Female CD1 mice were inoculated with ∼1.5×10^8^ CFU *S. aureus* Newman Sm^R^ or JSNZ Sm^R^ by intraperitoneal injection. Animal weights were monitored daily in Newman and JSNZ infected animals and are presented as percentage change from day 0 body weights (A). Bacterial loads in kidneys (B), liver (C) and spleen (D) were quantified on day 4 and compared using a two-tailed Mann-Whitney test. Day 4 kidney CFU *versus* weight change was plotted for JSNZ (E) and Newman (F) infected mice and a two-tailed Spearman correlation test applied. Results are combined from three independent experiments, each containing six mice per treatment group. Culture negative samples were plotted at the detection limit (dashed line). Median values are depicted.

Next, we determined the virulence of JSNZ in a renal abscess model (Fig. 2) [32]. Intraperitoneal inoculation of mice with ∼1.5×10^8^ CFU *S. aureus* JSNZ caused symptomatic disease with sustained weight loss of ∼15% on day 3 and 4. In contrast, Newman infected animals showed a transient weight loss of ∼10% on day 1, followed by a steady recovery of weight (Fig. 2A). Interestingly, JSNZ-infected animals had a significantly higher (30-fold) bacterial burden in the kidneys at day 4, whereas animals infected with Newman had a significantly higher (>20-fold) bacterial burden in the liver (Fig. 2B,C). The number of bacteria in the spleen was comparable (Fig. 2D). There was also a strong inverse correlation between body weight and kidney bacterial burden in mice infected with JSNZ (Fig. 2E) which was not evident in Newman infected animals (Fig. 2F). At a dose of ∼1.5×10^8^ CFU, JSNZ caused sustained symptoms of disease, particularly weight loss, in 78% (14/18) of animals. Increasing the dose to 2×10^8^ CFU led to 6/6 mice requiring euthanasia within 24 h of inoculation (data not shown).

**Figure 3:**
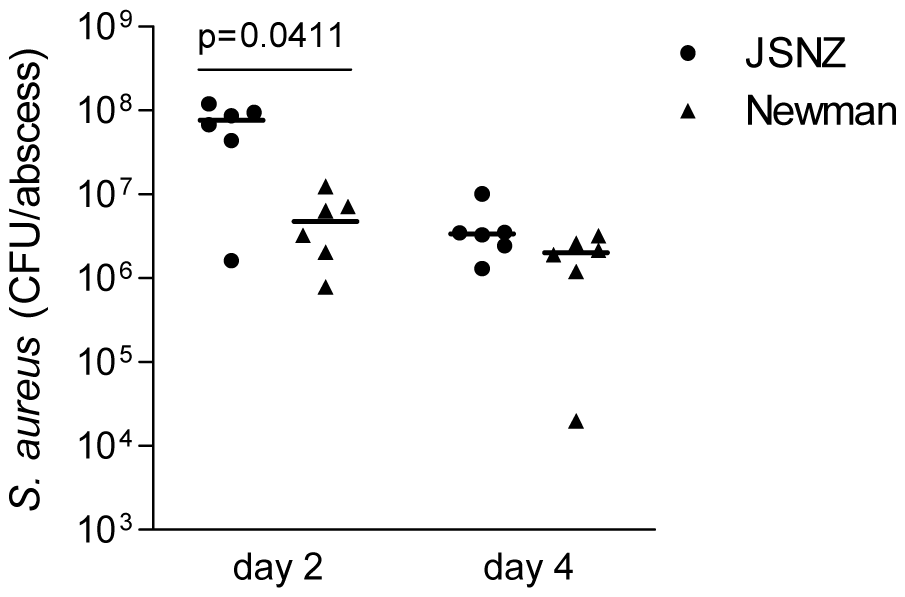
*S. aureus* JSNZ grows more rapidly than Newman after subcutaneous inoculation into mice. Female CD1 mice were inoculated subcutaneously on the right and left flanks with ∼5×10^6^ CFU *S. aureus* Newman Sm^R^ or JSNZ Sm^R^ in cytodex beads. Abscess tissue was harvested on days 2 and 4, the number of *S. aureus* per abscess enumerated and data compared using a two-tailed Mann-Whitney test. The result is representative of two independent experiments, each containing three or four mice per treatment group. Median values are depicted.

Finally, we investigated the virulence of JSNZ and Newman in a subcutaneous abscess model (Fig. 3) [30]. There were no differences in the appearance or development of abscesses throughout these studies. Abscess tissue from JSNZ-infected mice contained significantly higher (>15-fold) bacterial loads than Newman-infected tissue on day 2 post inoculation, whereas bacterial numbers were equivalent by day 4 (Fig. 3). Bacterial loads on day 4 were also comparable after inoculation with a lower dose of JSNZ or Newman (5×10^3^ CFU) (data not shown).

### JSNZ can be genetically modified by phage transduction and electroporation

Trans formation of many clinical *S. aureus* isolates with foreign DNA is efficiently blocked by restriction modify cation systems [33]–[35], which hamper their genetic manipulation. Interestingly, JSNZ could be easily genetically modified by phage transduction and electroporation (Table S1). JSNZ could be as effectively transduced as Newman and the restriction-defective laboratory strain RN4220. Similarly, the electroporation efficacy of JSNZ was comparable to Newman.

## Discussion

Within the staphylococcal research community, the current dogma is that mice are not natural hosts of *S. aureus* [10], [15]–[17]. This work describes a novel mouse-adapted *S. aureus* strain called JSNZ, isolated from our animal facility after the strain caused prolonged infection throughout a colony of C57BL/6J mice. Interestingly, JSNZ lacks any of the *hlb*-converting phages, which is a common theme among animal isolates [25], [27], [36]. Moreover, JSNZ was a found to be a better colonizer of mice and more virulent in an intraperitoneal infection model than the human-derived strain Newman, which is commonly used in mouse models of *S. aureus* disease. Finally, the strain could be easily genetically modified. JSNZ is the first mouse-adapted strain to be identified and studied in detail and could become an important tool to elucidate *S. aureus* host interactions in the mouse model.

An obvious lack of communication between laboratory animal veterinarians and staphylococcal researchers has led to the assumption that rodents are not natural hosts of *S. aureus*. However, in contrast to the general perception in the staphylococcal research community [10], [15]–[17], natural *S. aureus* colonization of laboratory mice, even under SPF conditions, is relatively common [18], [19] (Personal communication K. Pritchett, Charles River). Epidemiological studies are scarce, but according to textbooks on laboratory animal health, *S. aureus* is carried asymptomatically on the skin, the nasopharynx, and lower digestive tract of mice [18], [19]. For example *S. aureus* skin colonization was detected in 36% of C57BL/6 SPF mice [37]. Similarly, 9 out of 24 (37.5%) C57BL/6J breeding pairs at our animal facility were colonized in the nose and GI tract without showing clinical symptoms. Following the initial outbreak of preputial gland abscesses, breeders efficiently passed the strain on to their offspring for more than 2.5 years. Notably, JSNZ was not found in CD1 and BALB/c mice bred in the same areas as the infected C57BL/6J mice, demonstrating that good hygiene in animal facilities can prevent the spread of *S. aureus*.

While colonization might be common, *S. aureus* infections among laboratory rodents are generally considered to be rare [18], [19]. Typical clinical manifestations in mice include skin and soft tissue infections, e.g. abscesses, necrotizing dermatitis, furunculosis, and (in certain mouse strains) eye infections [18], [19], [38]. The JSNZ outbreak particularly affected male mice causing preputial gland adenitis, while female mice were generally symptom-free. Preputial gland abscesses, although infrequently investigated, seem to be sporadically common among male mice, and *S. aureus* is the most frequent causative agent [19], [39]. Overall, further studies are urgently needed to elucidate the epidemiology of natural *S. aureus* colonization and infection in mice.

Frequent *S. aureus* colonization of laboratory mice would have far reaching consequences not only for staphylococcal research and vaccine development, which require immunologically naïve mice, but also for mouse experimentation in general. Researchers usually do not screen their mice for *S. aureus* prior to experimental infection. Importantly, *S. aureus* is not on the list of pathogens excluded from SPF facilities and might therefore be frequently found even in SPF mice [37]. Thus, it would be advisable to include *S. aureus* in SPF screening programs.

The molecular characterization of JSNZ revealed an MLST type ST88 and a lack of the *hlb*-converting Sa3int phages, which can encode a human-specific immune evasion cluster [31]. ST88 strains are generally rare among human colonizing and hospital isolates [26],[40],[41], however, ST88 community-acquired methicillin-resistant *S. aureus* (CA-MRSA) infections have been recently reported from some parts of the world [42]–[44]. To identify molecular correlates of host-specialization, we compared the virulence gene repertoire of JSNZ with that of five closely related human ST78/88 methicillin-sensitive *S. aureus* strains. The *hlb*-converting Sa3int phages were absent in JSNZ, but detected in all five human-adapted CC88-strains. Similarly, microarray analysis revealed that 96% (138/143) of human CC88 strains from around the globe harboured a Sa3int phage (Stefan Monecke, unpublished data). This suggests that phage-encoded genes are crucial for infection and/or colonization of humans but not required in the murine host. In contrast, artificial colonization of human volunteers demonstrated that Sa3int phages are not essential in the first stages of *S. aureus* nasal colonization [25]. Nevertheless, this does not rule out an involvement of Sa3int phages in persistent human colonization and infection.

JSNZ also lacked the beta-lactamase gene cluster, which is commonly found in human strains and was detected in four out of five human CC88 isolates. This may imply limited exposure to beta-lactam antibiotics as is the case in animal breeding facilities. Apart from the Sa3int phage and the beta-lactamase genes, microarray analysis did not reveal any further differences between JSNZ and the human ST78/88 strains. The array design is based solely on human *S. aureus* isolates and thus may miss unique genes or gene variants specifically targeting the murine host. In line with this, recent genome analyses of bovine, ovine and poultry adapted strains point to a genetic pool of host-specific mobile genetic elements, including phages and pathogenicity islands [20]–[22]. To identify putative mouse-adapted mobile elements, we are currently sequencing the JSNZ genome.

Nasal carriage is an established risk factor for *S. aureus* infection, both in the hospital and in the community, with individuals often being infected with their own strain [6]. Moreover, *S. aureus* carriers pose an ongoing risk for the transmission of bacteria to susceptible individuals in health care settings [8]. Hence, there is a strong need for a robust and sustained mucosal colonization model to understand both bacterial and host factors that are important for establishing and maintaining colonization. Our data suggest that JSNZ could serve as a valuable tool to study *S. aureus* colonization in mice. Firstly, JSNZ is an excellent colonizer of laboratory mice. The strain persisted within a SPF C57BL/6J breeding colony for 2.5 years, colonizing the nose and GI tract without inducing clinical symptoms. Similarly, upon intranasal inoculation JSNZ induced robust and sustained colonization of the nose and GI tract of outbred CD1 mice. Secondly, while previous intranasal models have required continuous administration of streptomycin to knock down the endogenous flora and establish sustained colonization [10], [29], JSNZ was capable of competing with the endogenous flora for space and nutrients and to establish itself in these niches. As a matter of course, colonization models which do not require the application of antibiotics will reflect the clinical situation more closely. Thirdly, JSNZ spreads from the nose to the GI tract within a day, inducing persistent colonization of the gut mucosa. In this respect, human and murine colonization patterns seem to be very similar [4]. Human intestinal colonization has important clinical implications, including additional routes of transmission and the opportunity to acquire new antibiotic resistance genes, e.g. from vancomycin-resistant enterococci [4]. In a preliminary transmission experiment using co-housed colonized and naïve mice, we observed that JSNZ transmitted well to the nose and gut of two out of four naïve mice within three days, whereas Newman was not able to consistently colonize the co-housed littermates within the five days tested (data not shown). Finally, preliminary data show that the majority (53/104 strains) of murine *S. aureus* isolates obtained from laboratory mice in the United States belong to the MLST CC88 and are closely related to JSNZ (unpublished results). This suggests that ST88 could be the predominant murine *S. aureus* lineage.

Our findings suggest that JSNZ induces robust and prolonged mucosal colonization and is more virulent than Newman in a renal abscess model. Determining the molecular basis for this difference is challenging, because the strains are genetically very different. JSNZ and Newman belong to *S. aureus* lineages CC88 and CC8, respectively, and thus display lineage-specific variants of surface protein and regulatory genes as well as different mobile genetic elements. Even though JSNZ showed enhanced fitness in mice, relatively high inoculation doses were still required. Future work will therefore focus on the development of murine colonization and infection models which require lower inoculation doses and simulate the human clinical situation more closely, including natural colonization by transmission and colonization-to-disease models, where invasion is triggered in colonized mice by stress or immune suppression [45], [46].

In addition to JSNZ, two other potentially mouse-adapted *S. aureus* strains have been described in the literature. The *S. aureus* strain DAK was isolated following an outbreak of murine mastitis and colonized mice at rates comparable to Newman [29]. However, no further studies were conducted to characterize this strain in more detail. The *S. aureus* LS-1 strain caused an outbreak of septic arthritis and osteitis in NZB/W/B mice [47]. This toxic shock syndrome toxin (TSST)-1 producing strain was employed to study the pathogenesis of *S. aureus* arthritis in the mouse model, but again was never characterized in detail on a molecular level.

The alarming high incidence of illness and death within hospitals and the community caused by the bacterium *Staphylococcus aureus*, and the multidrug resistant nature of many isolates, has spurred efforts to develop vaccines and novel antibiotics [1], [48]. Their development and testing however, relies heavily on animal infection models that closely mimic the human clinical situation. Many researchers now routinely use clinical *S. aureus* isolates, i.e. USA300, in addition to commonly used laboratory strains such as Newman [49]–[51]. However, these isolates vary in their ability to cause disease in mice, depending on the genetic make-up of the isolate and the inoculation route [51], [52]. Indeed, there is no clear consensus over which clinical strain to use and many basic microbiological and immunological questions might be better answered using a standardized and well characterized *S. aureus* strain. We propose that JSNZ could serve as a valuable tool for studying bacterial and host factors involved in staphylococcal colonization and infection in the mouse model. Whether the strain is also suitable for studying *S. aureus* infections, other than subcutaneous and renal abscesses, remains to be clarified.

In summary, until now mice have not been regarded as natural hosts for *S. aureus* by the staphylococcal research community. We provide, for the first time, a detailed characterization of a mouse-adapted *S. aureus* strain, which caused an outbreak of disease in an animal breeding facility and subsequent long term colonization. With its enhanced ability for colonization and infection in the mouse and the feasibility of genetic manipulation, JSNZ could serve as a useful experimental tool to study bacterial and host factors involved in staphylococcal colonization in the mouse model, and for assessing the efficacy of novel vaccines and therapies directed at preventing colonization.

## Supporting Information

Table S1 **Complete DNA microarray results for JSNZ and human CC88 isolates.**
(XLSX)

Table S2 ***S. aureus*** **JSNZ can be easily genetically modified by phage transduction and electroporation.** ^a^A *lux*-encoding plasmid from *S. aureus* SA113 was transduced into various *S. aureus* strains using a Φ11 lysate. Results are given as median number of transductants with range for four technical replicates. ^b^A RN4220-derived plasmid was electroporated into various *S. aureus* strains. Results are given as median number of transformants with range, compiled data from three experiments, except ^c^ which were performed once). **Method S1 Transformation by electroporation and generalized phage transduction:** Electro-competent *S. aureus* cells were prepared by inoculating 100 ml TSB with *S. aureus* cells at A600 of 0.05 from a TSB overnight culture. The culture was grown at 200 rpm and 37°C to A600 of 0.8–1.0. Cells were harvested and washed twice in ice-cold sterile 0.5 M sucrose with 0.5 and 0.25 times the original culture volume. Afterwards, cells were placed on ice for 30 min to leach out the internal ion pool. After two more washing steps with 0.1 and 0.01 times the original culture volume, cells were split into 0.1 ml aliquots and frozen at −80°C. Electroporation was performed in 0.1 cm cuvettes at 100 Ω, 2500 V and 25 uF using a Biorad GenePulser XcellTM. 0.5 M sucrose in BHI medium was added directly into the cuvette immediately afterwards. After incubation at 37°C for 2 h without shaking, bacteria were plated onto TSA plates with selective antibiotics. 1 ng plasmid DNA (*E. coli* – *S. aureus* shuttle vector pUNKD) was electroporated into restriction-defective laboratory strain RN4220, laboratory strain Newman, JSNZ and several clinical isolates. Transformants were selected on TSA with erythromycin. Phage Φ11 was used to produce a phage lysate of *S. aureus* RN4220 containing an *E. coli*-*S.aureus* shuttle vector encoding the *lux* genes and GFP (pUNKD PFDH luxGFP, unpublished). The lysate was filtered through a 0.2-µm-pore-size filter and used to infect RN4220, Newman, JSNZ and several clinical isolates. Transductants were selected on TSA with erythromycin and screened for bioluminescence using an IVIS Kinetic™.
(DOCX)
