# Supplementary material for: Characterization of a Mouse-Adapted Staphylococcus aureus Strain
Source: PLoS One. 2013 Sep 2;8(9):e71142. doi: 10.1371/journal.pone.0071142 (PMC3759423; doi:10.1371/journal.pone.0071142)
Supplement: Table S2 — S. aureus JSNZ can be easily genetically modified by phage transduction and electroporation. aA lux-encoding plasmid from S. aureus SA113 was transduced into various S. aureus strains using a Φ11 lysate. Results are given as median number of transductants with range for four technical replicates. bA RN4220-derived plasmid was electroporated into various S. aureus strains. Results are given as median number of transformants with range, compiled data from three experiments, except c which were performed once). Method S1 Transformation by electroporation and generalized phage transduction: Electro-competent S. aureus cells were prepared by inoculating 100 ml TSB with S. aureus cells at A600 of 0.05 from a TSB overnight culture. The culture was grown at 200 rpm and 37°C to A600 of 0.8–1.0. Cells were harvested and washed twice in ice-cold sterile 0.5 M sucrose with 0.5 and 0.25 times the original culture volume. Afterwards, cells were placed on ice for 30 min to leach out the internal ion pool. After two more washing steps with 0.1 and 0.01 times the original culture volume, cells were split into 0.1 ml aliquots and frozen at −80°C. Electroporation was performed in 0.1 cm cuvettes at 100 Ω, 2500 V and 25 uF using a Biorad GenePulser XcellTM. 0.5 M sucrose in BHI medium was added directly into the cuvette immediately afterwards. After incubation at 37°C for 2 h without shaking, bacteria were plated onto TSA plates with selective antibiotics. 1 ng plasmid DNA (E. coli – S. aureus shuttle vector pUNKD) was electroporated into restriction-defective laboratory strain RN4220, laboratory strain Newman, JSNZ and several clinical isolates. Transformants were selected on TSA with erythromycin. Phage Φ11 was used to produce a phage lysate of S. aureus RN4220 containing an E. coli-S.aureus shuttle vector encoding the lux genes and GFP (pUNKD PFDH luxGFP, unpublished). The lysate was filtered through a 0.2-µm-pore-size filter and used to infect RN4220, Newman, JSNZ and several clini [file pone.0071142.s002.docx]

|  | RN4220  (ST8) | Newman  (ST8) | JSNZ  (ST88) | A17  (ST45) | A30  (ST5) | M15  (ST30) |
| --- | --- | --- | --- | --- | --- | --- |
| Transduction^a^ | 412.5  (237-540) | 427  (391-568) | 492.5  (302-840) | 0  (0-1) | 49.5  (5-106) | 41  (0-147) |
| Transformation^b^ | 385,000  (370,000-400,000) | 1200^c^ | 27,200  (2,000-27,600) | 0^c^ | 120^c^ | 0^c^ |
